# Immunomodulatory effect of *Syphacia obvelata* in treatment of experimental DSS-induced colitis in mouse model

**DOI:** 10.1038/s41598-019-55552-6

**Published:** 2019-12-13

**Authors:** Niloofar Taghipour, Nariman Mosaffa, Hamid Asadzadeh Aghdaei, Mohammad Rostami-Nejad, Joel V. Weinstock, Sarira Shahnavaz, Mohammad Reza Zali

**Affiliations:** 1 Basic and Molecular Epidemiology of Gastrointestinal Disorders Research Center, Research Institute for Gastroenterology and Liver Diseases, Shahid Beheshti University of Medical Sciences, Tehran, Iran; 2 Department of Parasitology and Mycology, School of Medicine, Shahid Beheshti University of Medical Sciences, Tehran, Iran; 3 Department of Immunology, School of Medicine, Shahid Beheshti University of Medical Sciences, Tehran, Iran; 4 Gastroenterology and Liver Diseases Research Center, Research Institute for Gastroenterology and Liver Diseases, Shahid Beheshti University of Medical Sciences, Tehran, Iran; 5 Division of Gastroenterology-Hepatology, Department of Internal Medicine, Tufts Medical Center, Boston, MA 02111 USA

**Keywords:** Immunotherapy, Immunosuppression

## Abstract

The ability of helminth parasite infections to manipulate the immune system of their host towards T regulatory responses has been proposed to suppress the inflammatory response. The aim of this study was to investigate the protective and therapeutic effect of *Syphacia obvelata* in the treatment of experimental DSS -induced colitis. 50 male C57BL/6 mice were divided into 5 groups: healthy uninfected controls, DSS colitis, receiving only *S. obv*, preventive (*S. obv* + DSS) and therapeutic group (DSS + *S.obv*). Colitis intensity was investigated by measuring body weight changes, stool consistency/bleeding and colon length. To evaluate the immune responses induced by this nematode, TNF-α, IL-10, IL-17, IFN-γ and expressing of FoxP3^+^ T cells were measured in mesenteric lymph nodes and Peyer’s patches cells. Mice in preventive and therapeutic groups treated with *S. obv* egg significantly ameliorated the severity of the DSS colitis, indicated by the reduced disease manifestations, improved histopathological scores correlated with the up regulation of Treg responses and down regulation of proinflammatory cytokines*. S. obv* can prevention and reverse on-going murine DSS colitis. The data suggest that induction of Tregs and change in cytokine profiles during helminthic therapies were responsible for reversed inflammatory events in IBD.

## Introduction

The inflammatory bowel disease (IBD) is a chronic immune diseases of the gastrointestinal tract often sub-divided into ulcerative colitis (UC) or Crohn’s disease (CD)^1–3^. The causes of IBD is unknown, but epidemiological and laboratory studies suggest that environmental and genetic factors associated with dysregulation of the mucosal immune system are important in the pathogenesis of IBD^4–6^. Approximately 400 million people in the world suffer from UC and/or CD^1–3^. The IBD prevalence in less developed nations is lower than in developed countries^7^. IBD is less frequent in rural compared to urban areas^8^ and less common in people exposed to various environmental pathogens and antigens^9^. These observations support the concept that environmental factors such as worm infections influence the prevalence of IBD^7^. The hygiene and old friends hypothesis are proposed to explain the rise in immunological disorders^3,10–15^. The hygiene hypothesis suggests that loss of exposure to helminths and other infectious agents during childhood increases susceptibility to immune disorders^10,16^. The old-friend’s mechanism argues that increase in chronic immune diseases consequence of loss of vital exposures to microorganisms that coexisted with humans throughout evolution^17,18^. Helminths seemed to be likely protective since it has long been appreciated that they modulate the host immune response, inducing immunologic tolerance via various mechanisms^19,20^. Thus, a comprehensive understanding of how helminths modulate host immunity may lead to novel, low risk and effective treatments for autoimmune diseases such as IBD. Ulcerative colitis and Crohn’s disease are treated with various medications in an attempt to control these diseases^3,21^. There is need for additional more effective novel therapies with fewer side effects, which could include helminth therapy^22^. There are many experimental and epidemiological studies supporting the concept that human parasitic infections block ongoing inflammation or prevent the development of IBD^3,23,24^. Helminths, specifically gastrointestinal (GI) nematodes, induce Treg, inhibit pro-inflammatory Th1 and Th17 responses and stimulate the production of regulatory cytokines like IL-10 and TGF-β which contribute to the protection from intestinal inflammation^25–28^. Also, GI nematode infection can induce regulatory dendritic cells that drive CD4^+^CD25^+^ FoxP3^+^ T cells (Tregs) development and the production of anti-inflammatory cytokines in the intestine and MLNs^25,29,30^. CD4^+^CD25^+^ Tregs are play an important role on nematode evasion from the host Th1-mediated attack and also play an essential function in regulating many immune responses and maintaining immune homeostasis^31–34^.

Various murine models of IBD are used to identify mechanisms of drug action and to test novel therapies. DSS-induced colitis is widely employed because the inflammation develops rapidly and is of predictable intensity^35–37^. Acute, chronic and relapsing models of intestinal inflammation can be produced by simply adjusting the concentration of DSS and the frequency of oral administration^35,37–39^.Various worms species such as *Trichuris muris*, *Trichuris suis* ova or larvae^40,41^, *Trichuris trichiura* ova^42^, *Necator americanus*^43^, *Heligmosomoides polygyrus bakeri*^44^ and *Hymenolepis diminuta*^45^ can prevent or reverse experimental colitis. There is a need to identify available and low risk worms suitable for therapeutic intervensions^46^. *Syphacia obvelata* is a pinworm that belongs to the order Oxyurina and lives in cecum and anterior colon of mice. The results of some studies using this nematode suggest it can modulate autoimmune diseases^46–48^.

This study investigated the potentiality use of *S.obvelata* as a novel therapy in IBD using DSS-induced colitis. The data show that *S. obvelata* prevents DSS-induced colitis and blocks ongoing inflammation. Moreover, they induce Tregs and modulate regulatory and pro-inflammatory cytokine expression in MLNs and PPs of their murine host.

## Material and Methods

### Experimental animals

Fifty parasite free C57BL/6 male mice aged 6–8 wk., weighing 20–24 g were purchased from the animal core facility at Royan Institute of Iran. All groups were matched by age, sex and body weight with a control group. The study was approved by ethic committee of Research Institute for Gastroenterology and Liver, Shahid Beheshti University of Medical Sciences, Tehran, Iran with ethic number: IR.SBMU. RIGLD.REC.1395.89 and we confirm that all methods were performed in accordance with the relevant guidelines and regulations.

From the first moments of the arrival of new mice from weaning phase, constant control of samples were begin. Therefore, all the mice during the study were free from nematode specially control groups and test groups. The location of the cages of the mice was completely separated and controlled by formalin-ether sedimentation and scotch-tape test^46^. After conducting a pilot tests, the cecal examination and scotch tape test revealed respectively the presence of worm and egg in C57BL/6 mice from the 12 and 14 days after eggs gavage. The chosen study duration was 28 days. Experimental mice were randomly divided into five groups (in our study, 5–6 mice in each group were required but in order to avoid bias and because of the risk of death in test groups, 10 mice per group were selected):

(1) *DSS-induced colitis*: Colitis was induced by oral administration of DSS (MW 36–50 kDa, MP Biomedicals, OH, USA), as described previously^35^. Briefly, all groups were deprived of drinking water for 2 h before the study. Mice received 2 cycles of 3% DSS for 4 day and 4 days of pure water between each cycle. Healthy control mice received normal drinking water.

(2) *S. obvelata infection group:* Infective *S. obvelata* eggs were propagated and maintained as described previously^46^*.* Mice received 500 infective *S. obvelata* eggs by oral gavage in 200 µl of 0.9% NaCl.

(3) *Preventive group (S. obv* + *DSS):* 12 days post *S. obvelata*-infection, the infected C57BL/6 mice received 16 days DSS induction.

(4) *Therapeutic group (DSS* + *S. obv):* 16 days after chemical DSS induction, mice was inoculated with 500 infective *S. obvelata* eggs via oral gavage and 12 days post DSS duration, mice were scarified.

(5) *Control group:* this group were matched by age, sex and body weight with test groups that received only water add libitum.

During the research period, body weight, general condition of health were monitored daily. On day 28, colon length was measured and of the distal colon was fixed in 4% formaldehyde for histological analysis. The severity of colitis was evaluate by following parameters:

### Disease activity index (DAI)

Throughout the research period, mice were observed daily for morbidity and given a DAI between 0 and 12 based on the following characteristic criteria: weight loss, fecal consistency, and occult/gross blood^49^. Occult blood was detected chemically using fecal occult blood clinical kits (SABA, Iran).

### Macroscopic and histopathological assessment

Spleen weight (measured in gram) and colons length (measured in cm) were determined. The entire colon was rapidly removed and separated from the cecum, then cleared from feces and blood by flashing with cold PBS. Small sections of distal colon were fixed in formaldehyde and prepared for histopathological examination. Sections were stained with hematoxylin & eosin and were examined in a blind manner by histopathologists^50^. The inflammation was scored by judging the degree of epithelial damage, inflammatory cells infiltration, crypt loss and goblet cells reduction^51^.

### Isolation and culture of MLNs and PPs lymphocytes

At the end of the treatment, the MLNs and PPs^52^ were removed and dissociated as previously described^46^. Briefly, the MLNs and PPs were dissociated in RPMI-1640 medium using sterile glass tissue grinders and filtered through a nylon cell strainer (Falcon; BD Labware, USA) to remove tissue debris. The dispersed cells were washed and suspended in complete tissue culture medium (RPMI, 10% FCS, 100 *μ*g of streptomycin/ml, 100 U penicillin/ml, 2 mM L-glutamine). Cells were cultured alone without stimulus in 24-well flat bottom plates at a concentration of 10^6^ cells/ml and incubated at 37 C, 5% CO2 for 12 h. The culture supernatants were collected and stored at −80 °C for cytokines assayed.

### Cytokines analysis

TNF-α, IFN-γ, IL-10 and IL-17 levels in the supernatant were measured by ELISA (R&D Systems, Minneapolis, MN, USA) according to manufacturer’s protocol. Briefly, these cytokines were detected by monoclonal anti-biotin antibody, which were evidenced by avidin-conjugated horseradish peroxidase followed by incubation with TMB substrate. Detection limits were 31.3 pg/ml for IL-10, IFN-γ and TNF-α; for IL-17 was 15.6 pg/ml. Measurements were done in triplicates, and the results were read at an OD of 450 nm using an Anthos ELISA reader (Anthos Labtech Instruments GmbH).

### Immunophenotyping (Flow cytometric) analysis

Flow cytometry was performed on harvested cells from the PPs and MLNs. Cells were stained using using a CD4^+^CD25^+^ Foxp3^+^ regulatory T cell staining kit (Miltenyi Biotech, Bergisch Gladbach, Germany). The following conjugated antibodies were incubated with cells samples: anti-mouse CD4- FITC, anti-mouse CD25- PE, and anti-mouse Foxp3 -APC. The cells were then analyzed by FACScalibur using Cell Quest Pro™ analysis software (BD Bioscience, USA), and data were analyzed by FlowingSoftware_2_5_1.

### Quantitative real-time PCR

Total RNA was extracted from the cells according to the YTA Total RNA Purification Mini kit (Yekta Tajhiz Azma, Iran) and was reversed transcribed using the RevertAid First Strand cDNA Synthesis Kit (Thermo Fisher Scientific, USA) according to the manufacturer’s instructions. Quantitative expression levels of Foxp3 and the reference gene (Mouse β-actin) were evaluated via SYBR Green I real-time PCR. Briefly, after synthesizing cDNA, real-time PCR was performed on Rotor_Gene6000 system (QIAGEN,Germany) using RealQ Plus 2x Master Mix Green (Ampliqon, Denmark) according to manufacturer’s protocols.

Each reaction contained 10 μL of 2x Master Mix, 0.5 μL of each specific primer **(**10 pm**/**l**)**, and 1 μL (5 ngr) of the cDNA, to a total volume of 20 μL. Reaction using the following thermal profile: 15 min at 95 ◦C, followed by 40 cycles of 20 s at 95 ◦C and 1 min at 58 ^◦^C. The primers used in PCR reactions were as follows: Foxp3 forward (5′-GGCCCTTCTCCAGGACAG-3′), Foxp3 reverse (5′- GCTGATCATGGCTGGGTTG -3′), and β-actin forward (5′-CTTCTTGGGTATGGAATCCTG-3′), β-actin reverse (5′- GTGTTGGCATAGAGGTCTTTAC -3′).

### Statistical analysis

All statistical analyses were performed using SPSS 16 and Graphpad Prism v6 software. Experimental values were given as the means ± SD. The statistical significance of any difference in each parameter among the groups was evaluated by One-way ANOVA followed by Bonferroni test. P value < 0.005 were considered significant.

### Ethical approval

This study received the approval from the Research Institute for Gastroenterology and Liver, Shahid Beheshti University of Medical Sciences, Tehran, Iran Ethical Committee with ethic number: IR.SBMU. RIGLD.REC.1395.89.

## Results

### Survival rates, DAI score, colon length

**Figure 1:**
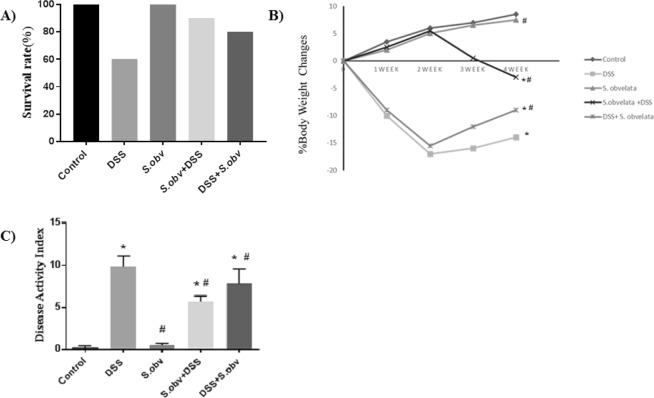
Treatment with *S. obvelata* reduced clinical signs of DSS-induced colitis in the C57BL/6 mice. (**A**) Survival rate in experimental period. (**B**) Weight change during trial, expressed as percentage change from day 0. C) Compare the DAI in all study groups. *Compared to the control group (P < 0/005). ^#^Compared to the DSS colitis group (P < 0/005).

*S. obvelata* infected colitis mice and control group significantly survived than colitis mice. We also found that survival rate in mice of the preventive and therapeutic group were significantly greater than those in the model group (Fig. 1A).

Remarkably, percentage body weight loss in control group (8.54 ± 0.42), and *S. obvelata* group (7.54 ± 0.59) were significantly lower than in DSS colitis mice (−14.58 ± 0.77). Mice body weight in therapeutic group (−8.82 ± 0.63) showed a more rapid improvement, compared with colitis group (Fig. 1B).

There was a significantly less scores in healthy control group (0.20 ± 0.21), and *S. obvelata* group (0.50 ± 0.22) than DSS-induced colitis group (9.84 ± 1.27). The DAI scores were significantly decreased in the preventive (5.68 ± 0.66) and therapeutic group (7.78 ± 1.78) than colitis group. Infection with *S. obvelata* reduced clinical signs of DSS colitis (Fig. 1C).

**Figure 2:**
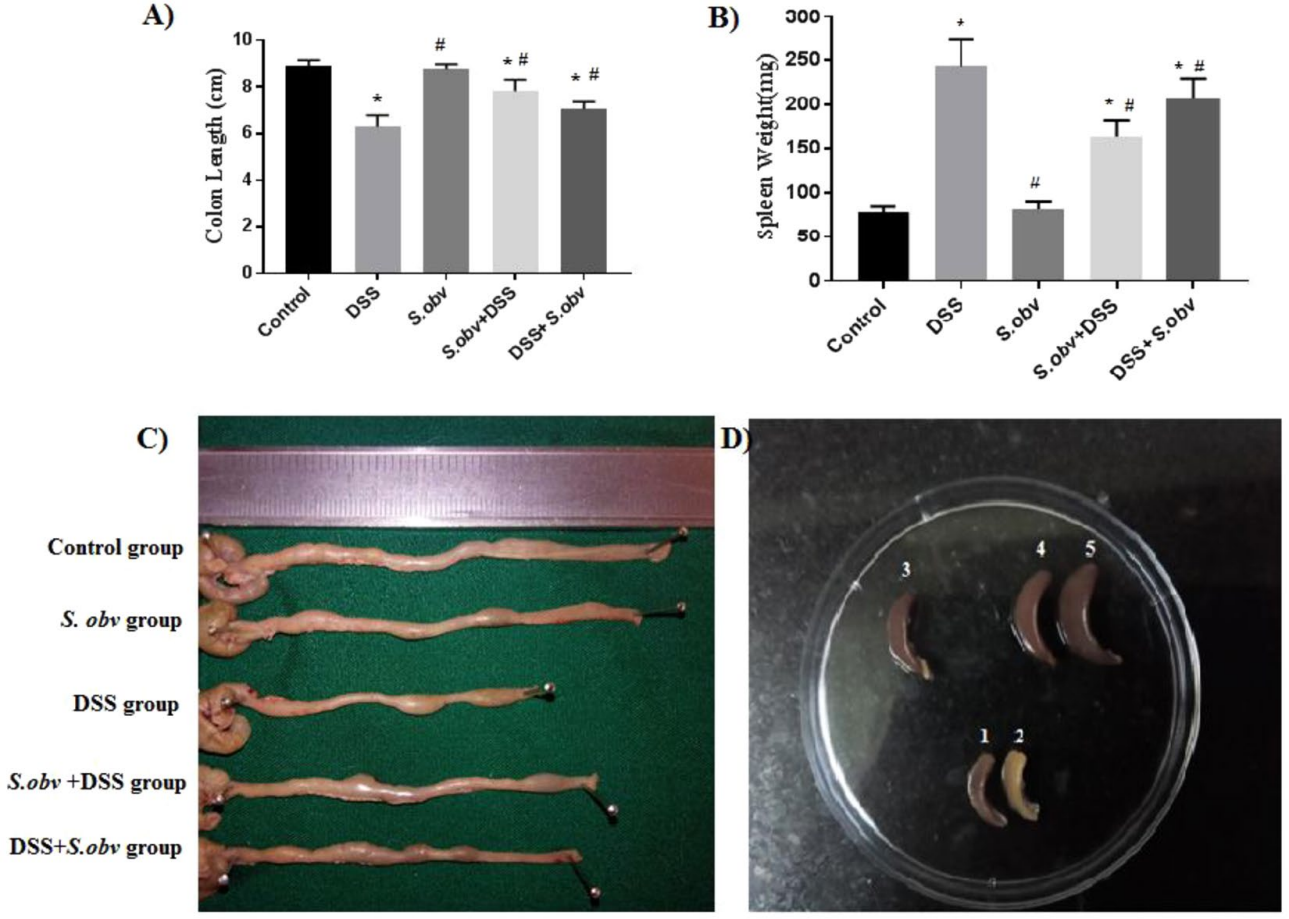
All mice were euthanized and scarified to remove the colons and spleens. (**A,C**) Compare the colons lengths in the experimental groups. (**B,D**) Compare the weight of the spleens in the study groups; representative samples are shown (1: *S. obv* group, 2: Control group, 3: *S.obv* + DSS group, 4: DSS + *S. obv* group, 5: *DSS* group). *Compared to the control group (P < 0/005). ^#^Compared to the DSS colitis group (P < 0/005).

Spleens weight among the studied groups were observed with significantly less scores in preventive (162.80 ± 20.36) and therapeutic group (206.60 ± 22.29) than DSS mice (243.68 ± 30.09) (Fig. 2A,C). Also, shortening the colon length among the experimental groups (Fig. 2) were rarely seen in preventive (7.82 ± 0.50) and therapeutic group (7.10 ± 0.32) than DSS mice (6.30 ± 0.50) (Fig. 2B,D). According to clinical manifestations, the results showed that the clinical presentation of colitis such as weight loss, rectal bleeding, spleen weight and colon length in all mice in the preventive and therapeutic group was significantly improved compared to the DSS model.

### Histopathological assessment

**Figure 3:**
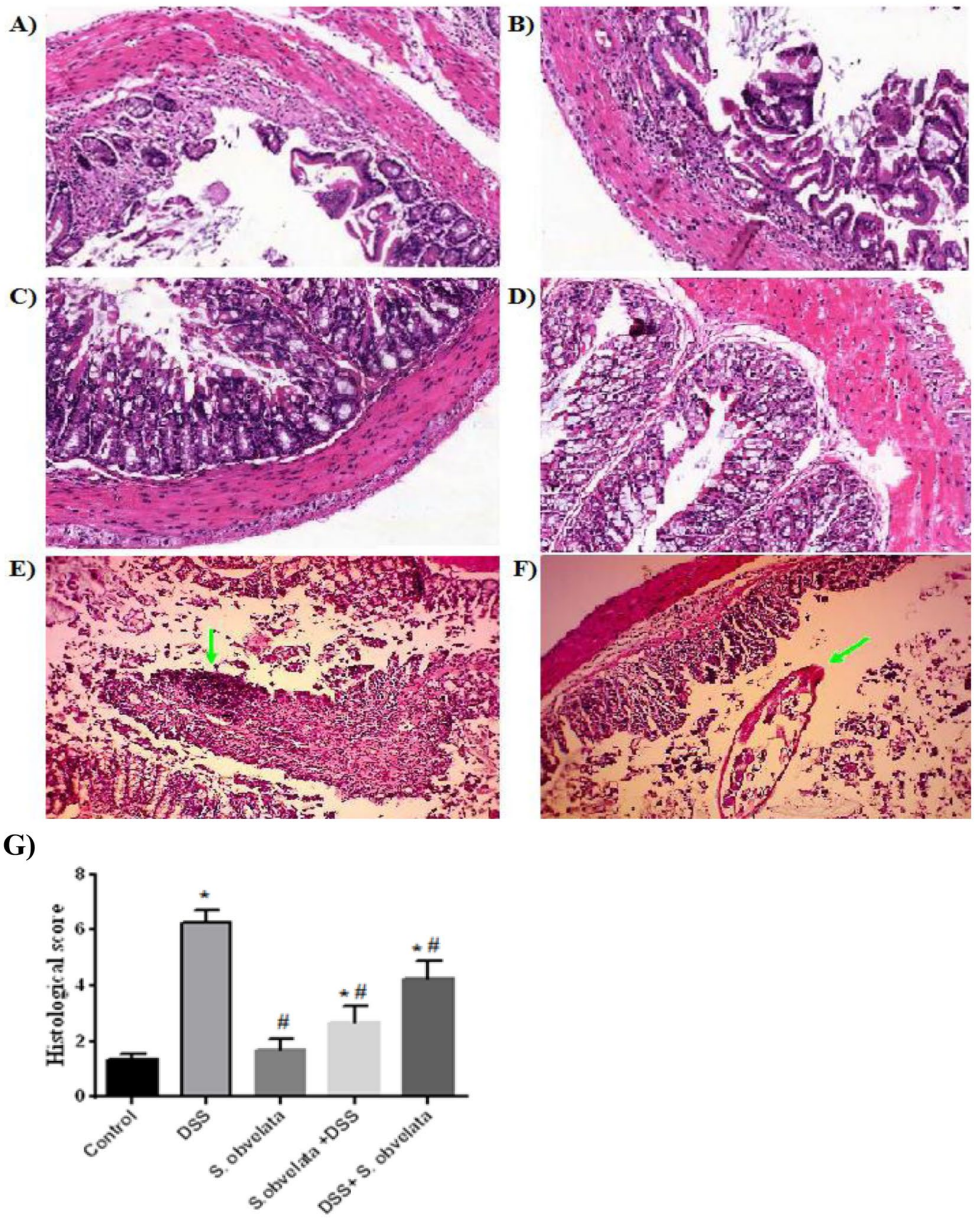
Histopathological results of colon sections with H&E stain in different study groups. (**A,B**) Visible loss of crypts and heavy inflammatory cellular infiltrate in colitis model. (**C**) Normal mucosa from healthy control. (**D**) Very few histological changes in *S. obv* group. (**E**) Moderate lymphocytic infiltration and loss of crypts in preventive group. (**F**) Relative improvement in colon tissue in the therapeutic group compared to the model group along with a cross sectional view of the nematode in the tissue.(**G**) Histological assessment in different study groups. *Compared to the control group (P < 0/005). ^#^Compared to the DSS colitis group (P < 0/005).

Based on histological assessments of the distal colon sections, healthy epithelium and normal thickness of the smooth muscle were observed in the control group (Fig. 3C). The least inflammation intensity (1.68 ± 0.41) were observed in the group exposed to *S. obvelata* only. The colons of *S. obv* group displayed no or minimal inflammation (Fig. 3D). The most increase of inflamation was observed in the DSS colitis group (6.28 ± 0.42) compared to the control group (1.34 ± 0.21). Loss of crypts, changes in the epithelial cell, decrease in the number of goblet cells and marked infiltration of inflammatory cells were seen in this group (Fig. 3A,B). Preventive (2.66 ± 0.59) and therapeutic (4.32 ± 0.71) groups showed a remarkably less pathological features, moderate inflammation and improvement in damaged tissue (Fig. 3E,F). In sight of these results, it was evident that *S.obv* infection ameliorated the severe inflammation of the colon induced by DSS (Fig. 3G).

### The immunophenotyping results of FoxP3^+^ Tregs staining

**Figure 4:**
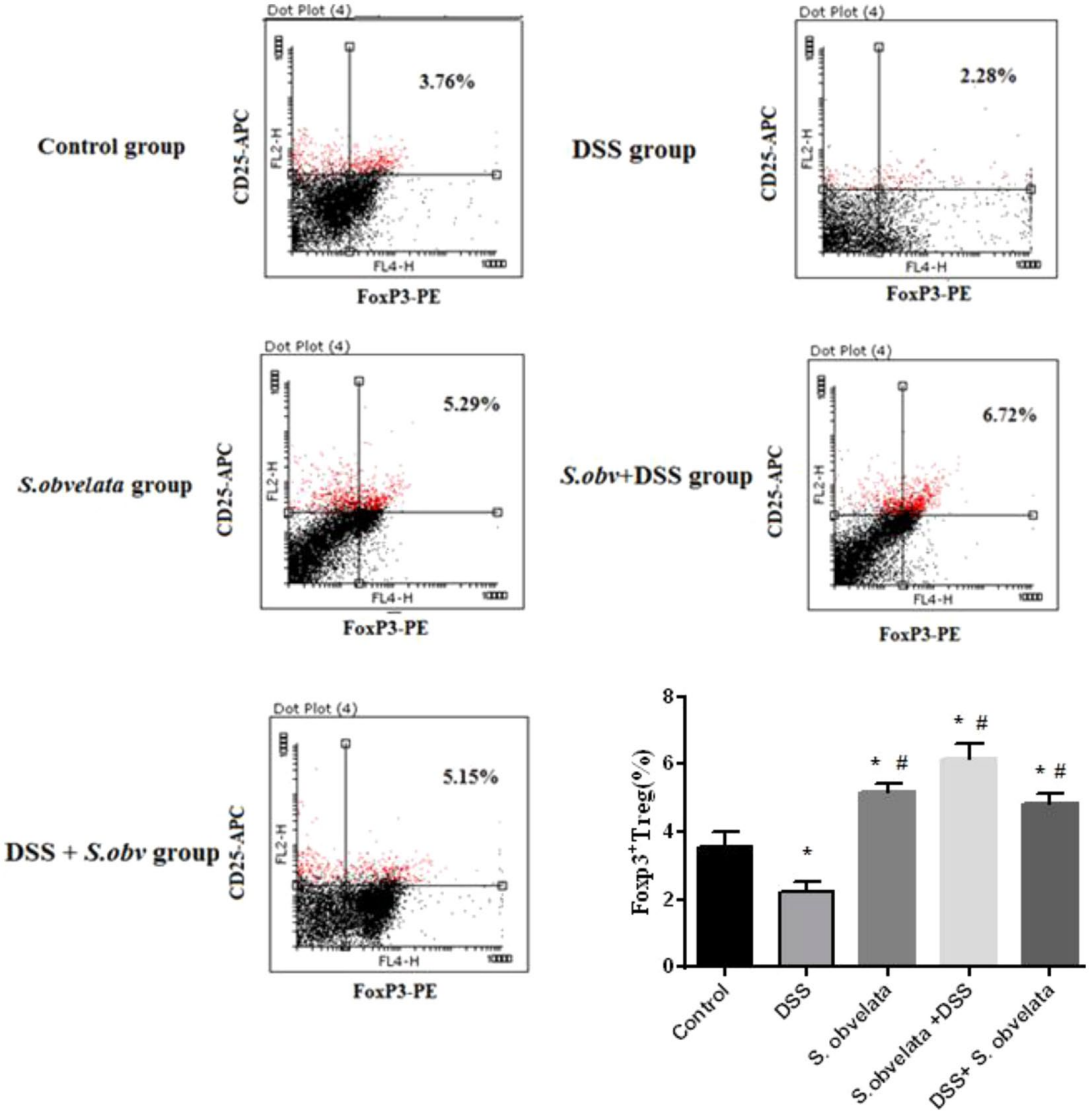
FACS analysis demonstrates the percentage of CD4^+^CD25^+^ FoxP3^+^ T regulatory cell in MLNs from a representative sample each group. Cell were stained with anti- CD4- FITC, anti- CD25- PE,anti- FoxP3 -APC. *Compared to the control group (P < 0/005). ^#^Compared to the DSS colitis group (P < 0/005).

**Figure 5:**
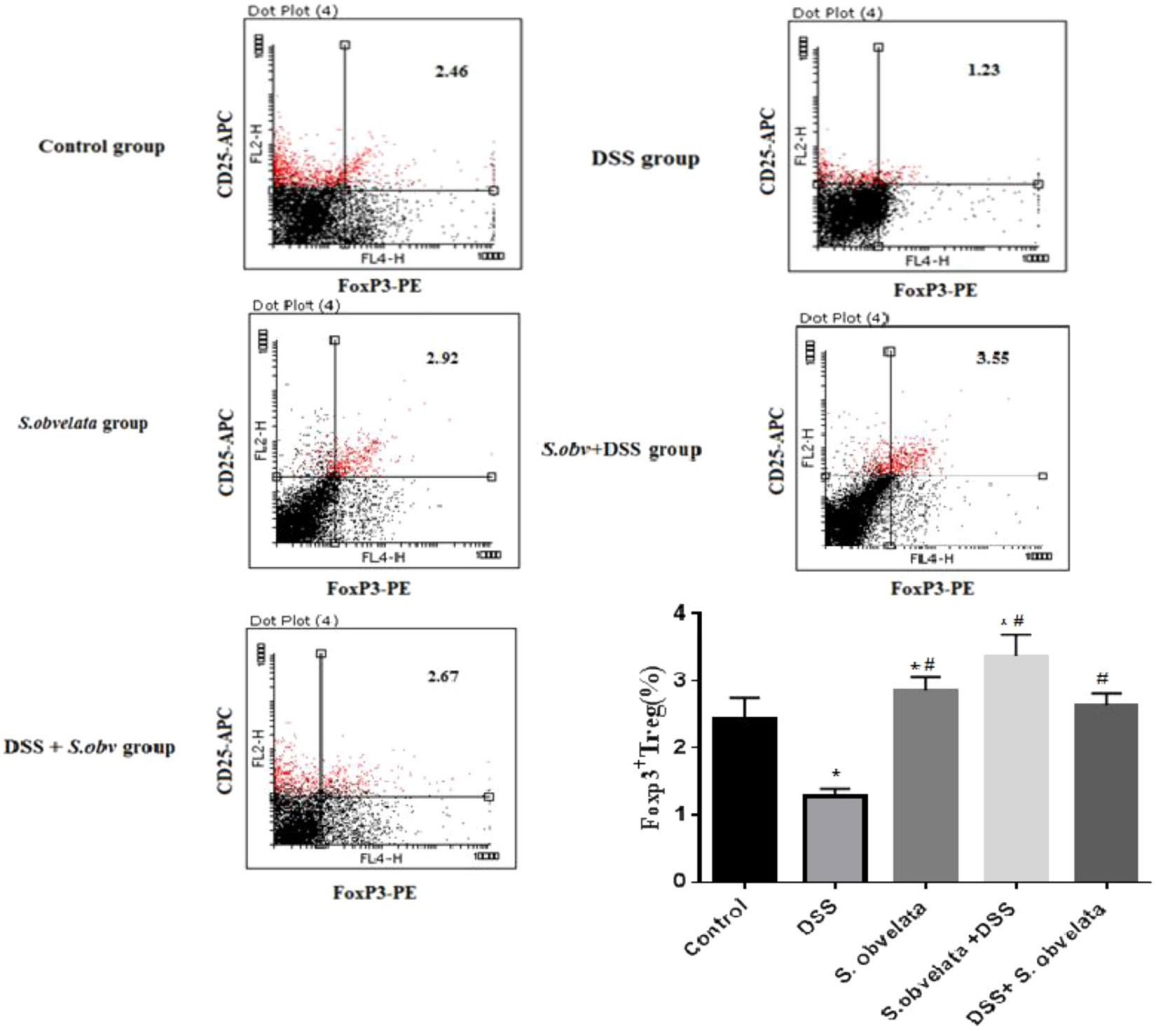
FACS analysis demonstrates the percentage of CD4^+^CD25^+^ FoxP3^+^ T regulatory cell in PPs from a representative sample each group. Cell were stained with anti- CD4- FITC, anti- CD25- PE, anti- FoxP3-APC. *Compared to the control group (P < 0.005). ^#^Compared to the DSS colitis group (P < 0.005).

Since Foxp3 is a specific marker for CD4^+^ CD25^+^ Tregs^53^, we determined the percentage of CD4^+^ CD25^+^ Foxp3^+^ T cells in all experimental group. There was a significant difference (p < 0.001) in the distribution of FoxP3^+^ T reg in MLNs and PPs among the different groups. In MLNs cells, Foxp3^+^ Treg peaked in preventive group (6.16 ± 0.48) followed by *S. obv* group (5.17 ± 0.27) and therapeutic group (4.86 ± 0.28) but barely noticed in control group (3.55 ± 0.46) and DSS colitis group (2.26 ± 0.26). The percentages of Foxp3^+^ Tregs in PPs lymphocytes in preventive group (3.37 ± 0.33) and *S. obv* group (2.87 ± 0.18) were significantly (p < 0.003) higher than those in control group (2.44 ± 0.31). Although increasing the percentage of FoxP3^+^ Treg was observed in therapeutic group (2.64 ± 0.17) compared to control group (2.44 ± 0.31), but this difference was not statistically significant. A significant reduction in Foxp3^+^ Treg in DSS colitis group (1.33 ± 0.27) was shown compared with the control group (2.44 ± 0.31). Representative dot plots of flow cytometry in all studied groups in MLNs and PPs cells are shown in Figs. 4 and 5 respectively.

### The ELISA assay

**Figure 6:**
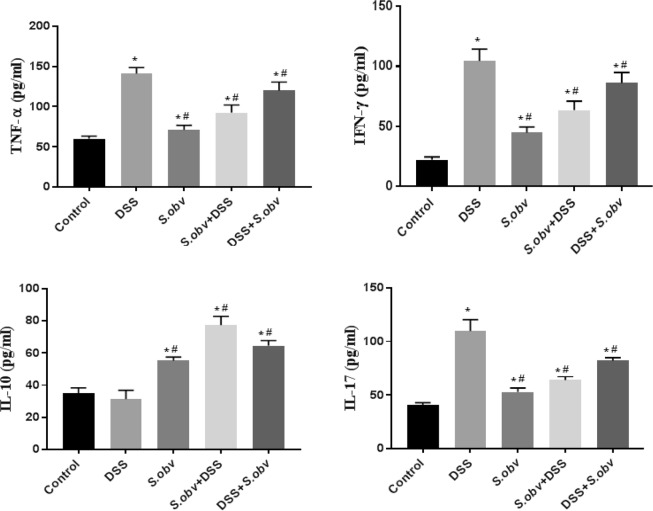
Effect of *S.obv* colonization on TNF-α, IFN-γ, IL-10 and IL-17 level in MLNs cell culture supernatants among different studied groups. *Compared to the control group (P < 0/005). ^#^Compared to the DSS colitis group (P < 0/005).

Figure 6 and Table 1 show production of cytokines by MLNs cells in the studied groups. The IL-10 production was increased in MLNs cells from preventive, therapeutic group and *S.obv* group compared with the control. The level of IL-10 secretion in the DSS group was lower than control, but this decrease was not statistically significant. The level of the pro-inflammatory cytokines such as TNF-α, IFN-γ, IL-17 in the DSS group was significantly increased compared to other groups. Production of this cytokines were increased in MLNs cells from preventive, therapeutic and *S.obv* group compared to controls and were significantly (P < 0/001) lower than DSS- receiving mice.

**Table 1:** Study of cytokine profiles among different studied groups in MLNs and PPs cell culture supernatants by ELISA.

Group	MLN	ANOVA		PP	ANOVA	
	Mean ± SD	F	p-value	Mean ± SD	F	p-value
**IL-10 (pg/ml)**						
Control	35.2 ± 3.2	110.58	0.001	44.9 ± 3.8	103.4	0.000
DSS	31.3 ± 5.6			50.2 ± 2.4		
*S. obvelata*	55.2 ± 2.2			85.5 ± 5.3		
*S.obvelata* + DSS	77.4 ± 5.4			113.3 ± 9.4		
DSS + *S. obvelata*	64.5 ± 3.3			95.6 ± 8.5		
**TNF-α (pg/ml)**						
Control	60.1 ± 3.3	92.12	0.001	40.5 ± 3.5	79.6	0.001
DSS	141.3 ± 7.3			97 ± 7.9		
*S. obvelata*	71.2 ± 5.8			48.2 ± 4.7		
*S.obvelata* + DSS	92.4 ± 9.7			59.3 ± 4.7		
DSS + *S. obvelata*	119.9 ± 10.8			74.9 ± 6.3		
**IFN-γ (pg/ml)**						
Control	22.3 ± 2.3	102.89	0 001	16.2 ± 0.3	116.5	0.001
DSS	104.5 ± 9.8			78.9 ± 3.9		
*S. obvelata*	44.8 ± 4.8			38.9 ± 5.3		
*S.obvelata* + DSS	63.5 ± 7.4			43.6 ± 4.6		
DSS + *S. obvelata*	86.1 ± 8.9			61.1 ± 7.4		
**IL-17 (pg/ml)**						
Control	41.1 ± 1.8	118.6	0.001	21.9 ± 2.8	111.8	0.001
DSS	109.8 ± 10.7			82.1 ± 6.3		
*S. obvelata*	52.9 ± 3.8			41.2 ± 2.5		
*S.obvelata* + DSS	64 ± 3.5			50.6 ± 5.2		
DSS + *S. obvelata*	82.1 ± 2.8			52.8 ± 5.18		

**Figure 7:**
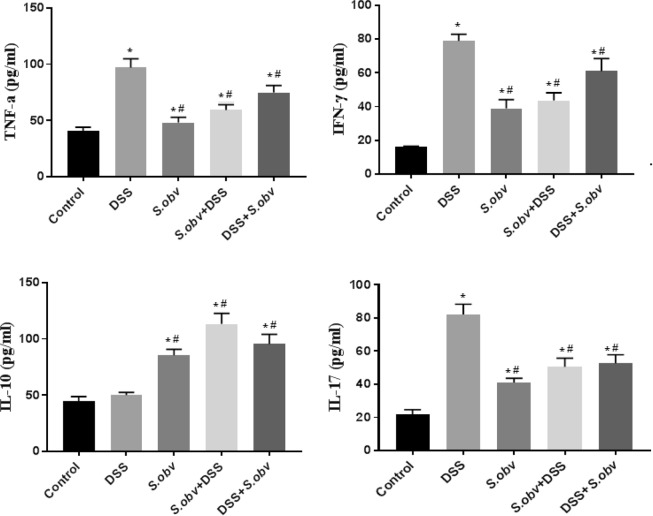
Effect of *S.obv* colonization on TNF-α, IFN-γ, IL-10 and IL-17 level in PPs cell culture supernatants among different studied groups. *Compared to the control group (P < 0/005). ^#^Compared to the DSS colitis group (P < 0/005).

Figure 7 and Table 1 display production of cytokines by PPs cells in the experimental groups. IL-10 production in colitis mice was significantly higher than control, but this increase was not statistically significant. The level of IL-10 were increased in PPs cells from preventive, therapeutic group and *S.obv* group compared with DSS model.

Production of IFN-γ, TNF-α, IL-17 cytokines were enhanced in PPs cells from therapeutic, preventive and *S.obv* model compared to controls and were significantly lower than DSS mice.

### Qualification of FoxP3 expression

**Figure 8:**
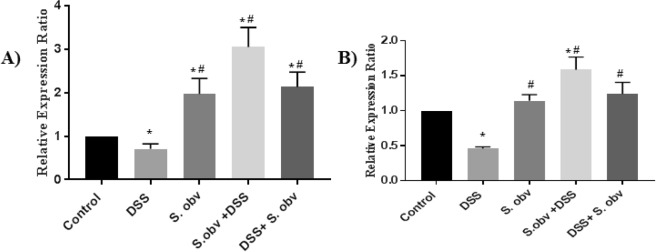
Comparison of the average FoxP3^+^ T regulatory cell count in (**A**) MLNs, (**B**) PPs in all studied groups. *Compared to the control group (P < 0/005). ^#^Compared to the DSS colitis group (P < 0/005).

qRT-PCR analysis was done to analyze the gene expression pattern of FoxP3 marker on cells isolated from the MLNs and PPs in mice treated with *S. obv* eggs (Fig. 8). This expression level in the group treated with DSS only was greatly lower than other groups. The Foxp3 expression level in *S. obv* and therapeutic groups in MLNs and PPs sites were slightly different and was significant higher than control and DSS models. In addition, the FoxP3 significantly increased in preventive groups compared to other groups. This study demonstrated that *S. obv* egg clearly drive the expression in cells isolated from MLNs and PPs sites. Up-regulation of FoxP3 mRNA expression in the preventive and therapeutic groups in MLNs cells were more visible than PPs cells. Real-time PCR data can be found in Supplemental file.

## Discussion

Inflammatory bowel disease (IBD) represents a group of idiopathic, chronic, globally-occurring gastrointestinal disorders and a main cause of disease and disability. IBDs have a dramatic rising incidence over the last two decades in the developed world^54^. Epidemiological, clinical trials and experimental studies support the idea that helminths could suppress immune-mediated chronic inflammation^11,25,55,56^. There is remarkable and strong evidence in mouse models and human trials that helminthic therapy with worm egg, excretory-secretory (ES) products, and helminth-derived synthetic molecules can provides novel opportunities for safer and more controllable therapeutics against chronic inflammatory diseases such as IBD^3,10–15,55^.

Helminths because of long-term survival in a host have immunosuppressive capabilities that can modulate the host immune responses^57,58^. This exposure, suppresses Th1 and Th17-related cytokines production, increases Th2-related cytokines and release of Treg -related cytokines (IL-10 and TGF-β), in many immunologic diseases^19,59^.

In the current study we describe the immunomodulatory effects of *S.obvelata* egg in murine DSS-colitis model for the first time. Many factors are involved about our choosing this helminth. The oxyurid nematode *S. obvelata* is a global outbreak among wild rodents and it is safe for humans around the enviruments. *S. obvelata* is easy to easy to collect, keep and cultivation worms and control the stages of reproduction and its complete development and *S. obvelata* does not invading the epithelial layer. It can be used in areas of human life where there is a prohibition of the use of swine products. *S. obvelata* is easily eradicated by anthelmintic drugs, such as piperazine, thiabendazole, mebendazole, or ivermectin.

So far, few research has been done on the immunological effects of this parasite. For example, Michels *et al*. have reported that *S.obvelata* infection induced protective response with elevated Th2 cytokines and reduced cytokine responses in Ovalbumin-Induced Allergic models^47^. Pearson *et al*. have showed that infected rats with *S.obvelata* have a reduced incidence of arthritis after injection of Freund’s complete adjuvant^48^. Also the result of our previous study confirmed that *S. obvelata* may increase the immunological suppressive function in the intestinal tract^46^.

In this study, immunization with *S.obvelata* egg significantly protected from the development of colitis. We should also point out that since the *S obvelata* is a common parasite of laboratory mice, Therefore researchers have to be very careful and respect the clean rules that any infection could interfere the experimental studies. In order to ensure the absence of parasitic infections in this study, always formalin-ether sedimentation and scotch-tape test were done.

DSS induces colonic tissue injury, diarrhea, bleeding and loss of body weight^49^. Rectal bleeding and body weight loss are associated with enlargement of spleen and shortening of the colon in the DSS-induced colitis group. The data show that body weight loss due to disease was mitigated by worm infection. Also, DAI, macroscopic and histopathological scores in the two tests groups harboring worm infection were considerably lower than in the DSS-induced colitis group. Colonic shortening and the effect of DSS on spleen weight were less in the worm-infected mice. Histological changes causes by DSS exposure included goblet cell depletion, epithelial erosion and crypt distortion, inflammatory infiltration. Worm infected decreased the severity of mucosal damage and the intensity of the inflammation in both the preventive and therapeutic groups.

Another goal of this research was to evaluate immunological changes in PPs and MLNs. These tissues have a central role in orchestrating induction of oral tolerance^60^. PPs are the primitive location of Tregs and MLNs are the location of Tregs education. Inflammation in colitis may be caused by the loss of homeostasis between FoxP3^+^ Tregs and proinflammatory cytokines^3^. Some studies have shown increased in Foxp3^+^Treg cell frequency in the colitis patients^55–57^ but in our and also another studies, the decrease in Treg population in colitis models and patients were observed^61^.

In this study we have investigated Treg cells and measured FoxP3 mRNA in PPs and MLNs of groups of study. We used surface expression of CD4^+^CD25^+^ with FoxP3 intracellular staining for investigation of Treg population. FoxP3^+^ Tregs were present in a significantly higher numbers in PPs and MLNs of mice from *S.obvelata* immunized group that received the egg as a prophylactic and therapeutic helminthic therapy against DSS- induced colitis compare to uninfected control and DSS exposed animals.

MLNs showed the strongest increase expression of the FoxP3 marker. These data suggest that Foxp3^+^ T cells have an important role in helminths protection against DSS induced colitis, although the issue was no addressed in this study.

The results of a similar study that investigated the effect of another worm infection on PPs and MLNs has shown somewhat different results. Mosconi *et al*. calculated Treg ratios in PPs and MLNs in mice infected by *H. polygyrus bakery*. They reported that due to the penetration of invasive larvae into the mucosal wall, the Treg accumulation in PPs was greater than of the MLNs population^62^. *H.polygyrus* larvae also promoted expression of Treg *in vitro* in dispersed PPs cells. Our study may have yielded different results because *S. obvelata* does not invading the epithelial layer.

The investigation also quantified cytokine production by dispersed PPs and MLNs cell maintained in culture. INF-γ and IL-17 implicated in driving the inflammation in several models of murine colitis and IL-10 inhibited the production of pro-inflammatory mediators such as INF-γ, TNF-α^3^ and limited the disease^63^. We observed reduced levels of TNF-α, INF-γ and IL-17 in the cell supernatants of PPs and MLNs from *S. obvelata* infected mice (preventive and therapeutic groups compare with the uninfected DSS group). Cells from mice in the preventive group secreted lower amounts of inflammatory cytokines compared to cells from the therapeutic group. IL-17 secretion was high in uninfected mice exposed to DSS.

Ruyssers *et al*.^64^, Hasby *et al*.^3^ and Watanabe *et al*.^65^ investigated the immunological effect of S*. mansoni* ova and proteins. They also proved that *S. mansoni* is effective against experimental colitis. Their results showed improvement of inflammation after treatment with *S. mansoni* might be linked to regulatory T cells, as they found significant up regulation of IL-10 mRNA expression in T cells isolated from colonic tissues of treated mice^64^. Their results suggested that natural development of a regulatory immune response and cytokine profiles are distinct in different intestinal lymphoid tissue types^64^. In present study, there was a significant positive correlation between average FoxP3^+^ T regulatory cells and IL-10 in preventive and therapeutic *S.obvelata* group and a significant negative correlation between IL-10 and INF-γ, TNF-α in the same group. The higher level of IL-10 was seen in preventive and therapeutic groups compared with DSS groups. The IL-10 level showed significant increase in PPs cultured cells compared with MLNs cells. This correlation between IL-10 and FoxP3^+^ Treg cells is coincides with other helminthic therapy studies^65–67^. IL-10 is important in regulating effectors responses emerging in response to infection^64,68–71^.

Therefore, we concluded that *S.obvelata* over the course of life and long-term contact with the gastrointestinal tract utilizes anti-inflammatory strategies to escape host immunity and induce intestinal tolerance^46^. A strong link has shown between *S.obvelata* long-term chronic infections and Treg cell activity. It appears that mice received *S.obvelata* like some nematodes can simulated tolerogenic-DCs (tDCs) and macrophages boosted Treg cells that secreted regulatory cytokines, such as IL-10 and TGF-β^72,73^, and inhibited the Th1- and Th17-related cytokines that leaded to attenuation of experimental colitis.

Our results proposed that the useful effect of *S.obvelata* egg and compounds is linked to stimulation of regulatory T cells and suppression of proinflammatory cytokines. Therefore, inhibition of inflammation by the *S.obvelata* egg indicates its potential for decreasing the risk of colitis. Finally we suggest that treatment with *S. obvelata* egg and its products have prophylactic and therapeutic potentiality effect for DSS-induced colitis model.

## Supplementary information

Dataset 1
